# Anti-EMT and anti-fibrosis effects of protocatechuic aldehyde in renal proximal tubular cells and the unilateral ureteral obstruction animal model

**DOI:** 10.1080/13880209.2022.2088809

**Published:** 2022-06-25

**Authors:** Yu-Teng Chang, Mu-Chi Chung, Chi-Hao Chang, Kuan-Hsun Chiu, Jeng-Jer Shieh, Ming-Ju Wu

**Affiliations:** a Institute of Biomedical Sciences, National Chung Hsing University, Taichung, Taiwan; b Division of Nephrology, Department of Internal Medicine, Taichung Veterans General Hospital, Taichung, Taiwan; c Department of Post-Baccalaureate Medicine, College of Medicine, National Chung Hsing University, Taichung, Taiwan; d Rong Hsing Research Center for Translational Medicine, National Chung Hsing University, Taichung, Taiwan; e Ph.D. Program in Translational Medicine, National Chung Hsing University, Taichung, Taiwan; f Department of Medical Laboratory Science and Biotechnology, Asia University, Taichung, Taiwan; g Department of Medical Research, Taichung Veterans General Hospital, Taichung, Taiwan; h School of Medicine, Chung Shan Medical University, Taichung, Taiwan; i Graduate Institute of Clinical Medical Sciences, School of Medicine, China Medical University, Taichung, Taiwan

**Keywords:** Epithelial–mesenchymal transition, renal fibrosis, unilateral ureteral obstruction

## Abstract

**Context:**

Protocatechuic aldehyde (PCA) is a natural product that has various benefits for fibrosis.

**Objective:**

This study evaluated the effects of PCA on renal fibrosis.

**Materials and methods:**

Epithelial–mesenchymal transition (EMT) was induced by 20 ng/mL transforming growth factor-β1 (TGF-β1), followed by treatment with 1 and 5 μM PCA, in the rat renal proximal tubular cell line NRK-52E. Cell viability, protein expression, and scratch wound-healing assays were conducted. Sprague–Dawley (SD) rats underwent unilateral ureteral obstruction (UUO) surgery for renal fibrosis indication and were treated with 50 and 100 mg/kg PCA for 14 days.

**Results:**

The IC_50_ of PCA was appropriately 13.75 ± 1.91 μM in NRK-52E cells, and no significant difference at concentrations less than 5 μM. PCA ameliorated TGF-β1-induced EMT, such as enhanced E-cadherin and decreased vimentin. Fibrotic markers collagen IV and α-smooth muscle actin (α-SMA) increased in TGF-β1-induced NRK-52E. Moreover, PCA reduced TGF-β1-induced migration in the wound-healing assay. Analysis of rat kidneys indicated that PCA reduced UUO-induced hydronephrosis (control: 15.11 ± 1.00%; UUO: 39.89 ± 1.91%; UUO + PCA50: 18.37 ± 1.61%; UUO + PCA100: 17.67 ± 1.39%). Protein level demonstrated that PCA not only decreased vimentin expression and enhanced E-cadherin expression, but inhibited UUO-induced collagen IV and α-SMA upregulation, indicating that it could mitigate EMT in a rat model of UUO-induced renal fibrosis.

**Discussion and conclusions:**

This study suggested that PCA decreases TGF-β1-induced fibrosis and EMT *in vitro* and *in vivo*. These findings demonstrate pharmacological effects of PCA and might be a potential strategy for the prevention of organ fibrosis in clinics.

## Introduction

Chronic kidney disease (CKD) is defined as the presence of kidney damage or decreased kidney function for ≥3 months. As CKD progresses, normal renal tissue is replaced by interstitial fibrotic tissue (Raman et al. 2014), which, in turn, damages normal tissue and prevents its regeneration and function.

Fibrosis is a normal bodily function that aids in wound-healing, tissue-remodeling, and is a protective mechanism in response to stress and injury. It is a repair process that maintains the original structural and functional integrity of the organ tissue (Lee and Kalluri 2010). However, during the fibrotic reaction, the effectiveness of normal healing is reduced, and continuous exposure to chronic injury leads to tissue fibrosis. Marked extracellular matrix deposition and scar formation accelerate fibrosis progression, ultimately leading to organ failure (Lee and Kalluri 2010; Rockey et al. 2015). The pathogenesis of renal fibrosis is a gradual process which can lead to end-stage renal failure (Liu 2006). Renal fibrosis includes inflammatory cell infiltration, tubular atrophy, renal interstitial fibrosis, and glomerulosclerosis (Eddy 1996). Among various profibrotic factors, transforming growth factor-β1 (TGF-β1) is a key protein responsible for fibrosis in most organs and causes renal epithelial cells to transform into fibroblasts. Epithelial–mesenchymal transition (EMT) causes cell diversification in complex tissues. This dynamic process helps organize the formation of organisms and is critical in the differentiation of various tissues and organs. In CKD, renal tubular cells lose their epithelial phenotype through EMT and transform into a mesenchyme phenotype, which then leads to fibrosis (Liu 2004).

Small molecules are one of the leading sources of bioactive compounds. Investigating bioactive compounds of natural products are imperative to disease therapy including fibrosis in multiple organs (Feng et al. 2020). Multiple bioactive compounds from natural products have already been identified with the potential to treat CKD and renal fibrosis (Chen et al. 2018). Discovering a bioactive small molecular that can affect one of these targets would subsequently inhibit its expression and fibroblast proliferation. Anti-fibrotic effects of Chinese herbal medicines were observed in kidney disease by inhibiting Wnt/β-catenin signalling such as Qishen Yiqi dripping pill (QYDP), and poricoic acid A (PAA) (Chen et al. 2020b; Li et al. 2021). In UUO mice, 5-methoxytryptophan (5-MTP) derived from l-tryptophan improves renal interstitial fibrosis by IκB/NF-κB signalling inhibition and Keap1/Nrf2 pathway enhancement (Chen et al. 2019). The metabolite 1-hydroxypyrene and 1-aminopyrene mediates renal fibrosis via regulation of aryl hydrocarbon receptor signalling pathway (Miao et al. 2020, 2022). Accumulating evidence has demonstrated that bioactive compounds could facilitate the drug development in anti-renal fibrosis.

PCA is a water-soluble phenolic acid compound isolated from a traditional Chinese herbal medicine. PCA has various pharmacological activities. These include, antiatherosclerosis by downregulating tumour necrosis factor-α and intercellular cell adhesion molecular-1 secretion (Zhou et al. 2005); anti-apoptosis through significantly inhibiting caspase-3 activity (Xing et al. 2012); antioxidation and anti-inflammatory by eliminating 2,2-diphenyl-1-picrylhydrazyl (DPPH) free radical scavenging and inhibiting nitric oxide (NO) production (Chang et al. 2011); protection of cardiomyocytes by suppressing endoplasmic reticulum (ER) stress-associated signalling pathways (Wan et al. 2021). PCA can effectively alleviate cisplatin-induced acute kidney injury (Gao et al. 2016). In fibrosis, PCA can inhibit TGF-β1 expression (Li et al. 2012), effectively reverse EMT, and prevent pulmonary fibrosis (Zhang et al. 2015). In hepatic fibrosis, PCA can significantly reduce the degree of fibrosis, effectively improve biochemical criteria and histopathological types associated with fibrosis and reduces TGF-β1 expression. PCA can also mediate anti-fibrosis and anti-EMT in diabetic mice kidneys (Chang et al. 2021).

In this study, we investigated whether PCA can reduce EMT and renal fibrosis. To achieve this, PCA was administered in the TGF-β1-treated rat renal tubular epithelial cell line, NRK-52E, and a rat unilateral ureteral obstruction (UUO) model.

## Materials and methods

### Materials and instruments

We obtained TGF-β1 recombinant protein from Sino Biological (80116-R08H, Beijing, China); Dulbecco’s modified Eagle’s medium (DMEM) from Gibco (Carlsbad, CA); foetal calf serum from Hyclone (Logan, UT); PCA extracted powder (#820475), and mouse polyclonal anti-GAPDH (MABT825) from Merck Millipore (Carrigtwohill, County Cork, Ireland); mouse monoclonal anti-E-cadherin (610182) from BD Biosciences (San Diego, CA); rabbit polyclonal anti-E-cadherin (20874-1-AP) from ProteinTech Group (Chicago, IL); rabbit polyclonal anti-vimentin (IR45-137), and rabbit polyclonal anti-α-SMA (IE47-146) from IReal Biotechnology (Hsinchu, Taiwan); rabbit polyclonal anti-collagen IV (ab6586) from Abcam (Cambridge, UK); horseradish peroxidase (HRP)-labeled anti-rabbit and anti-mouse secondary antibodies from Cell Signaling Technology (Topsfield, MA); Western imprinting equipment from Amersham Imager 680 (Cytiva, Marlborough, MA).

### Cell culture and treatment

The normal rat kidney tubular epithelial cell line, NRK-52E, was cultured in high-glucose DMEM containing 4 mM l-glutamine (Gibco), 5% foetal calf serum, and 1% antibiotic–antimycotic solution (Gibco). The cells were incubated in a humidified atmosphere under 5% CO_2_ at 37 °C. The medium was replaced every 2–3 days. Renal fibrosis was induced in cells by treatment with TGF-β1 (PeproTech, Cranbury, NJ) for 7 days.

### Cell viability

Cells were cultured in 24-well plates at 8 × 10^2^ per well and incubated with TGF-β or PCA for the indicated experiments. The medium was changed every 3 days. After 1 week, the medium was discarded, and the cells were washed with phosphate-buffered saline (PBS). Cell counting kit-8 (CCK-8, MedChemExpress, Monmouth Junction, NJ) measuring solution was added into each well and incubated for 4 h at 37 °C. The plates were then assayed using a microplate reader at 450 nm.

### Western blotting

The total protein was isolated using the following method. Cells were harvested, lysed in radioimmunoprecipitation assay (RIPA) lysis buffer (Millipore), homogenized, and centrifuged. The supernatant was then boiled in a sample buffer with an aliquot corresponding to 50 μg of protein separated through sodium dodecyl sulfate–polyacrylamide gel electrophoresis (SDS-PAGE) and transferred onto polyvinylidene difluoride (PVDF) membranes (Merck Millipore). The membranes were blocked with 5% non-fat milk for an hour then incubated with diluted primary antibodies (1:2000) for 18–20 h at 4 °C. Next, the membranes were washed and incubated HRP-labeled diluted secondary antibodies (1:10,000) at room temperature for 1 h and then detected using Amersham Imager 680 (Cytiva). All data were presented in at least three independent experiments and were utilized ImageJ software (National Institutes of Health, Bethesda, MD) for quantitation.

### Wound healing

NRK-25E cells were seeded into 6-well culture plates. After the specified amount of time, a scratch wound was made with the tip of a 1 mL pipette at the centre of the plate. The cells were washed with PBS to remove floating cells, incubated for 16 h in a serum-free conditioned medium, and then analyzed and photographed through a microscope (NIKON, Ti-U, Tokyo, Japan); the distance was analyzed using a scale bar.

### Immunofluorescence staining

The cells were fixed in 4% paraformaldehyde for 15 min and incubated in 1% Triton X-100 for 15 min at 25 °C. The cells were then incubated with the primary antibody, E-cadherin (1:500) or α-smooth muscle actin (α-SMA) (1:500), overnight at 4 °C, followed by incubation with the secondary antibodies, Alexa Fluor 488 goat anti-rabbit and Alexa Fluor 546 goat anti-mouse, for 1 h at 25 °C. The coverslips were mounted in DAPI Fluormount-G (SouthernBiotech, Birmingham, AL) and scanned with laser-scanning confocal microscopy (FLUOVIEW FV1000, Olympus, Central Valley, PA).

### Animal model

Male Sprague–Dawley (SD) rats (∼200 g) were obtained from the National Laboratory Animal Centre (Taipei, Taiwan). The rats were divided into four groups (*n* = 6 per group): (1) sham–vehicle, where animals underwent sham operations and were treated with the vehicle; (2) UUO–vehicle, where the animals underwent UUO and were treated with vehicle, as described previously (Wu et al. 2006); (3) UUO–PCA50, where the rats underwent UUO and were treated with PCA (50 mg/kg/day); and (4) UUO–PCA100, where the rats underwent UUO and were treated with PCA (100 mg/kg/day). The PCA dosage chosen was based upon a previously published study (Xu et al. 2012). The wound was closed in layers. The rats were maintained in a temperature-controlled room, and their vital signs were monitored regularly after surgery. PCA was dissolved in sterilized water and administered by oral gavage every day. The efficacy of PCA therapy on UUO-induced renal fibrosis was examined on day 14. All animal care and experimental procedures were approved and conducted by the Committee for Animal Experiments, Taichung Veterans General Hospital, Taiwan (approved document La-1061475). The PCA was delivered by oral gavage since UUO surgery for 14 days. The used concentration was referred to the other studies of PCA treatment in SD rats (Xu et al. 2012).

### Immunohistochemical staining

After kidney tissues were fixed in 10% formalin, the lumens were inspected for grossly visible lesions. All immunohistochemical studies were performed on paraffin-embedded sections. The paraffin-embedded kidney sections were deparaffinized in xylene, hydrated in graded alcohol and water, and subsequently placed in 3% H_2_O_2_ to eliminate endogenous peroxidase activity. Next, the sections were blocked with normal goat serum and incubated with the primary antibodies overnight at 4 °C. As a negative control, the primary antibody was replaced with normal rabbit IgG, and staining was performed. All staining methods followed the protocols of Dako Cytomation EnVision + Dual Link System-HRP (DAB+) (Dako Cytomation Inc., Carpenteria, CA). For morphometric analysis, the sections were stained with periodic acid–Schiff (PAS). Masson’s trichrome staining was used to demonstrate collagen deposition. All slides were scanned using an Axiovert 200 M (Zeiss, Jena, Germany) and quantified using NIS-elements BR software 4.0.

### Statistical analysis

Data are presented as mean ± standard error of the mean. Student’s *t*-test was used to compare the differences between the control and experimental groups. GraphPad Prism version 6.0 (GraphPad Software, La Jolla, CA) was used for statistical analysis.

## Results

### Toxicity analysis of TGF-β1 and PCA in NRK-52E

**Figure 1.**
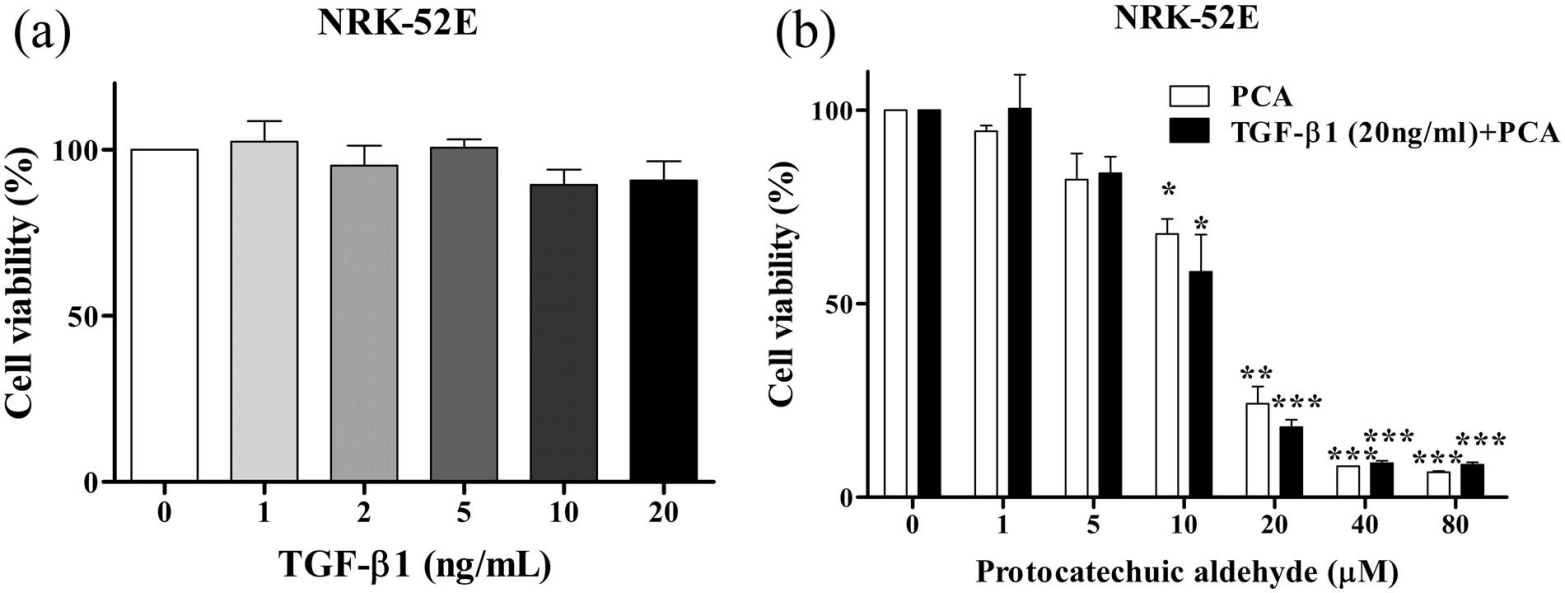
Effect of PCA and TGF-β1 on the viability of NRK-52E cells. (a) Cells were exposed to various TGF-β1 concentrations for 7 days, as detected by the CCK-8 assay. (b) Dose-dependent effects of PCA with and without exposure to 20 ng/mL TGF-β1 on NRK-52E cell viability for 7 days. Data are presented as mean ± SEM of three independent experiments. *p < 0.05, **p < 0.01, and ***p < 0.001 compared with each group control.

The toxicity dosage of TGF-β1 was examined. NRK-52E was treated with TGF-β1 at 0, 1, 2, 5, 10, or 20 ng/mL. There are no significant differences were observed after treatment on cell viability (Figure 1(a)), thus, 20 ng/mL TGF-β1 was used for the remaining experiments. Next, to investigate if PCA affects the growth in NRK-52E cells, different concentrations of PCA (0, 1, 5, 10, 20, 40, and 80 µM) were administered. PCA at concentrations ≥10 µM significantly reduced cell viability (IC_50_: 13.75 ± 1.91 µM). Cotreatment with TGF-β1 and various concentrations of PCA also caused cell viability to be significantly decreased when PCA concentrations were ≥10 µM (Figure 1(b)). Thus, PCA concentrations ≤5 µM were used in this study.

#### Effects of PCA on EMT induced by TGF-β1 in NRK-52E

**Figure 2.**
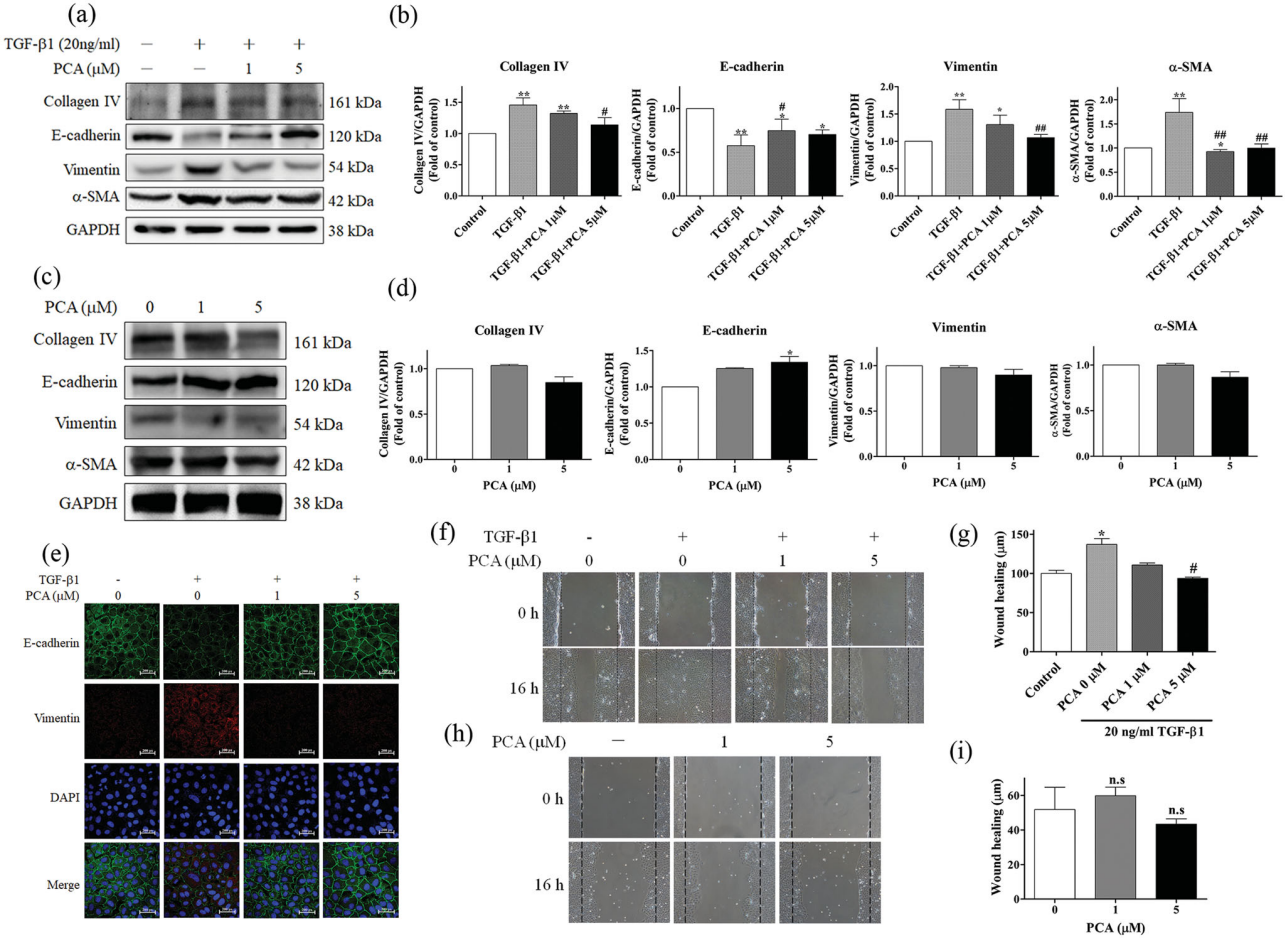
PCA decreased TGF-β1-induced EMT in NRK-52E cells. The experiment was divided into four groups: control, TGF-β1 only, TGF-β1 and 1 µM PCA, and TGF-β1 and 5 µM PCA. (a) Western blot revealed EMT-related protein expression. (c) PCA treated with 0, 1, and 5 µM in NRK-52E cells. Western blotting analysis of EMT-related protein expression. (e) Immunofluorescence was used to observe the EMT markers E-cadherin, vimentin, and DAPI which is a nuclear stain. Scale bar represents 200 µm. (f) To analyze the migration distance, the cells were treated with 0, 1, or 5 µM PCA with TGF-β1 (20 ng/mL) for 7 days and then performed the wound-healing assay for 16 h. (h) The cells were then treated with 0, 1, or 5 µM PCA alone and observed under a microscope. (b, d, g and i) The quantified results from (a, c, f, and h), respectively. Data are presented as mean ± SEM of at least three independent experiments. *p < 0.05, and **p < 0.01 compared with control. #*p* < 0.05, and ##*p* < 0.01 compared to the TGF-β1 group.

NRK-52E cells were divided into four groups: control (no treatment), treatment with TGF-β1 alone, cotreatment with TGF-β1 and 1 µM PCA, and cotreatment with TGF-β1 and 5 µM PCA. The expression of EMT markers (E-cadherin, vimentin, and α-SMA) and fibrosis markers (collagen IV) was evaluated through Western blot analysis. As presented in Figure 2(a) and quantified in Figure 2(b), cotreatment with 1 or 5 µM PCA and TGF-β1 significantly attenuated TGF-β1-induced EMT and fibrosis by inhibiting the expression of collagen IV, vimentin, and α-SMA protein but increasing E-cadherin expression. As compared with control, the PCA treatment only groups showed mild increasing in the protein expression of E-cadherin, but no significant change in other fibrosis markers (Figure 2(c,d)). 

In immunofluorescence staining, cotreatment with 1 or 5 µM PCA and TGF-β1 increased E-cadherin expression but decreased vimentin expression (Figure 2(e)). NRK-52E cells were treated with TGF-β1 alone or with PCA (1 or 5 µM) for 7 days and then performed the wound healing assay for 16 h. The results (Figure 2(f,g)) revealed that the distance of wound healing in the TGF-β1-only group was significantly higher than that in the control group (*p* = 0.0322). Cotreatment with TGF-β1 and 5 µM PCA significantly decreased the wound-healing distance compared with TGF-β1 alone (*p* = 0.0206). There was no significant change observed in the PCA treatment only groups (Figure 2(h,i)). Taken together, these results indicate that PCA inhibits TGF-β1-induced fibrosis and EMT in NRK-52E cells.

#### Effects of PCA on rat renal tubulointerstitial changes and functions induced by UUO surgery

**Figure 3.**
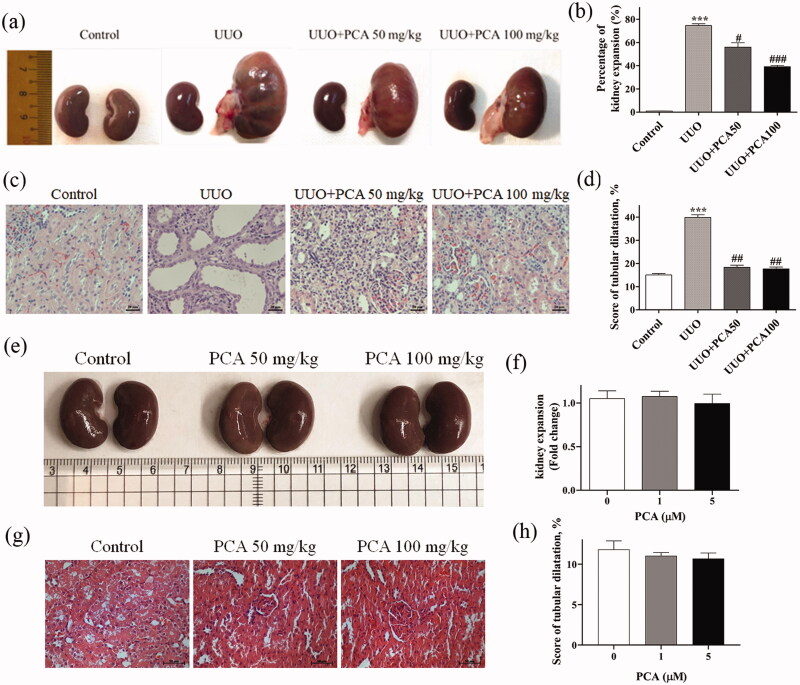
The volume and pathology of rat kidneys after UUO surgery and PCA treatment. SD rats were subjected to UUO surgery to induce fibrosis, and oral gavage with PCA was performed every day. After 14 days, kidney volumes were observed and quantified. (a) Rat kidney volume for four groups; the kidney on the right side of each picture is the left kidney. (b) Quantification of kidney expansion of the left kidney compared to the right kidney. (c) H&E staining of the renal tissues in each treatment group. PCA decreased tubular dilation induced by UUO in rat renal tissues. Scale bar represents 20 µm. (d) Quantification of H&E stain for analyzed tubular dilution (e) Representative pictures of kidney isolated from various dosage of PCA groups and quantified in (f). (g) Pathological damage was analyzed by H&E staining in SD rats with 1 or 5 µM PCA treatment and capture by inverted microscope and quantified in (h). Scale bar represents 50 µm. (f and h) The quantified results from (e) and (g), respectively. Data are presented as the mean ± SEM of at least three independent experiments. *p < 0.05, **p < 0.01 and ***p < 0.001 compared with control. #*p* < 0.05, ##*p* < 0.01, ###*p* < 0.001 compared to UUO rats.

SD rats were subjected to UUO surgery to induce renal fibrosis, followed by feeding and oral gavage with PCA. Two PCA concentrations were chosen, 50 and 100 mg/kg. SD rats were divided into four groups: Sham (sham surgery, i.e., control group), UUO, UUO + 50 mg/kg PCA (UUO + PCA50), and UUO + 100 mg/kg PCA (UUO + PCA100). At 14 days after surgery, the rats were sacrificed, the sizes of the kidneys with or without ureteral ligation in each group were compared (Figure 3(a)) and quantification of kidney expansion of the left kidney compared to the right kidney was presented by percentage (control: 1.00 ± 0.13%; UUO: 74.60 ± 2.75%; UUO + PCA50: 56.01 ± 6.58%; UUO + PCA100: 39.18 ± 1.71%) (Figure 3(b)). The kidney size in the UUO group was significantly higher than that in the control group (*p* < 0.001). Additionally, the UUO group kidney size was significantly smaller in the UUO + PCA50 group (*p* = 0.0208) and in the UUO + PCA100 group (*p* < 0.001), indicating that PCA can effectively inhibit the degree of kidney expansion caused by UUO surgery. The SD rat kidneys were examined under a microscope using haematoxylin and eosin (H&E) staining. For H&E staining results (Figure 3(c,d)), the degree of renal tubular dilatation was significantly higher in the UUO group than in the control group (control: 15.11 ± 1.00%; UUO: 39.89 ± 1.91%; UUO + PCA50: 18.37 ± 1.61%; UUO + PCA100: 17.67 ± 1.39%). It was also significantly lower in the UUO + PCA50 and UUO + PCA100 groups than in the UUO group. There are no significant changes observed in the kidney size and kidney structure in the PCA treatment only group (Figure 3(e–h)).

#### Effects of PCA on renal fibrosis and EMT changes in rats after UUO surgery

**Figure 4.**
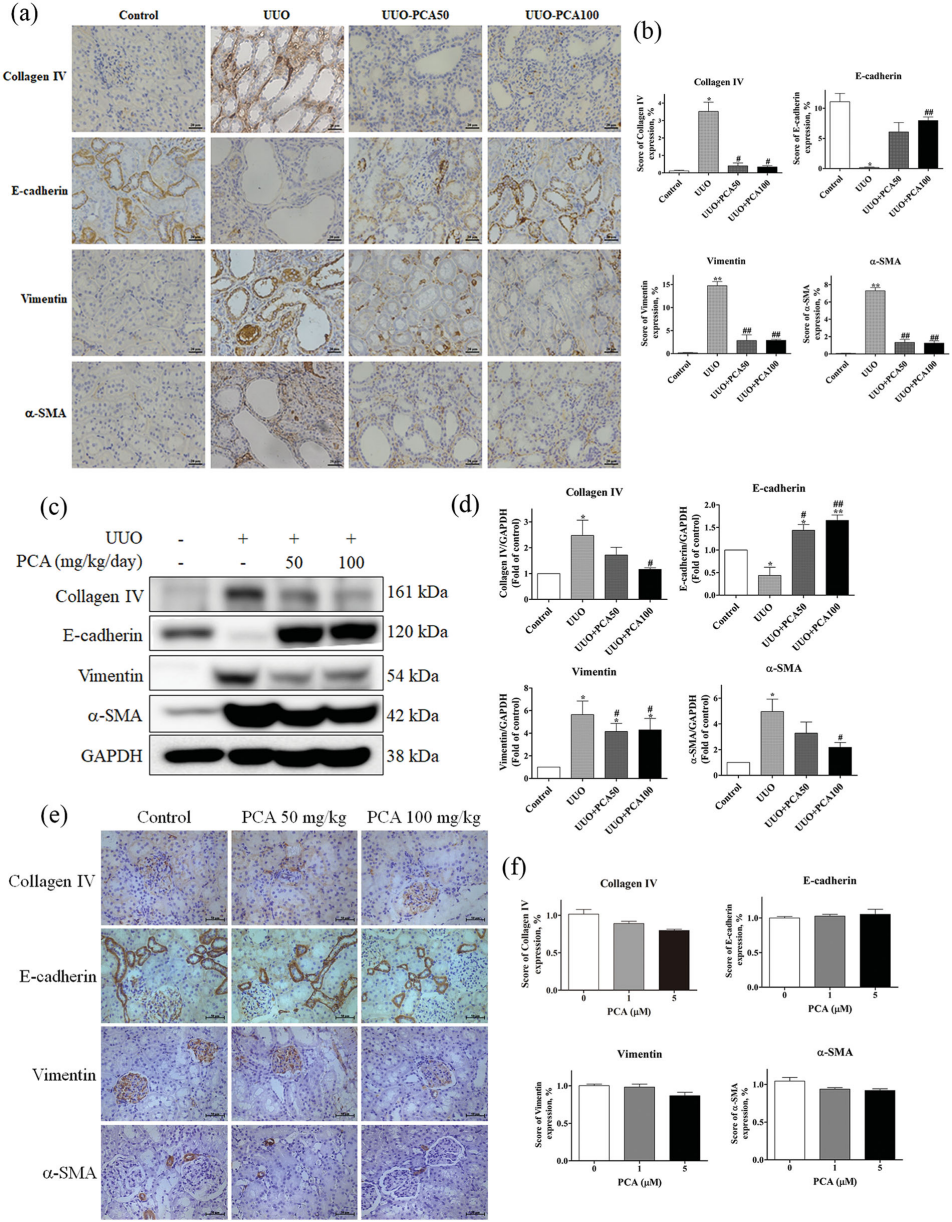
PCA decreased EMT induced by UUO in rat renal tissues. (a) Fibrosis and EMT markers were observed through immunohistochemistry. Scale bar represents 20 µm. (b) Quantification of immunohistochemistry results from (a). (c) EMT- and fibrosis-related proteins were evaluated by Western blotting. (d) Quantitative analysis of Western blotting from (c). (e) Photographs of immunohistochemistry for EMT-related proteins in the kidney of rats with 0, 1, or 5 µM PCA treatment. Scale bar represents 50 µm. (f) The immunohistochemical intensity of (e) was quantified. Data are presented as the mean ± SEM of at least three independent experiments. *p < 0.05, **p < 0.01, and ***p < 0.001 compared with control. #*p* < 0.05, ##*p* < 0.01, ###*p* < 0.001 compared to UUO rats.

The expression of EMT epithelial cell marker protein E-cadherin, the EMT interstitial cell marker proteins, vimentin and α-SMA, and the fibrosis-related protein collagen IV in UUO rats with and without PCA treatment were examined using immunohistochemistry staining and were quantified (Figure 4(a,b)). As shown in Figure 4(a), E-cadherin expression was significantly lower in the UUO group than in the control group and significantly higher in the UUO + PCA100 group than in the UUO group. The expression of vimentin, α-SMA, and collagen IV was significantly higher in the UUO group than in the control group. The expression levels of vimentin and α-SMA were significantly lower in the UUO + PCA50 and UUO + PCA100 groups than in the UUO group. Taken together, these data indicate that PCA could prevent tubulointerstitial damage in rat kidney tissue on day 14 after UUO surgery.

Next, we used Western blot analysis to confirm the alteration in EMT-associated protein expression (Figure 4(c,d)). Compared with the control group, the UUO group exhibited decreased E-cadherin expression and increased expression of vimentin, α-SMA, and collagen IV. Compared with the UUO group, UUO + PCA50 and UUO + PCA100 groups exhibited increased E-cadherin expression but decreased vimentin, α-SMA, and collagen IV expression. However, the protein expression was not changed in PCA treatment only groups were observed (Figure 4(e,f)). These results indicate that PCA may exert protective effects against renal fibrosis.

## Discussion

TGF-β1 is a critical profibrotic cytokine and induces EMT (Yoshioka et al. 1993; Isaka 2018), which leads to fibrosis. Thus, TGF-β1 is an essential drug target for fibrosis (Ng et al. 1998; Le et al. 2005). Many studies have demonstrated the direct role of TGF-β in fibrotic kidney disease, including upregulation of TGF-β signalling in glomeruli or tubulointerstitium in the fibrotic kidney, kidney fibrosis induction by increased TGF-β, and amelioration of kidney fibrosis by anti-TGF-β therapy. Increased TGF-β expression was also observed in progressive glomerular diseases in humans and the fibrotic areas of biopsy specimen were strongly correlated with TGF-β1 expression (Yoshioka et al. 1993; Isaka 2018). Studies focusing on TGF-β1 activity in renal fibrosis models (such as UUO) (Isaka 2018) have revealed that TGF-β1 and EMT can be targeted for the treatment of renal fibrosis.

In our *in vitro* study, TGF-β1 was used to induce EMT and fibrosis in NRK-52E cells, which were inhibited by PCA. The EMT response increases the capability of cells to migrate (Liu 2004). In our experiment, PCA decreased TGF-β1-induced wound healing. These results demonstrated that PCA can reduce TGF-β1-induced EMT and cell migration in NRK-52E cells.

In our *in vivo* study, renal fibrosis was induced in male SD rats through UUO surgery, and PCA treatment inhibited the degree of renal swelling. Our data indicates that PCA can effectively inhibit renal fibrosis in UUO rats. However, we observed differences in renal tubules and stroma. Since ureteral obstruction occurs due to UUO surgery. Reflux and accumulation can result in tubular dilation which damages tubular cells and leads to transdifferentiation, which causes fibrosis. H&E and immunohistochemical staining revealed that the renal tubules were significantly dilated in the UUO group compared with the control group. In the UUO + PCA groups, this dilation was inhibited compared to the UUO group. UUO surgery is a well-characterized hydronephrosis model featured by interstitial inflammatory cell infiltration and tubular dilatation, followed by tubular interstitial fibrosis that blocks the kidney (Wu et al. 2006). PCA has been found to elicit antioxidant and anti-inflammatory behaviours in diabetic mice kidneys (Chang et al. 2021), these might be the reason that UUO rats can reduce fibrosis and tubular dilatation through PCA treatment.

Maintaining mitochondrial homeostasis provides sufficient energy to support renal function. However, mitochondrial dysfunction was observed in the renal cells of patients with acute kidney injury (AKI) and diabetic nephropathy (Bhargava and Schnellmann 2017). Moreover, the crosstalk between (NADPH oxidases) NOXs and mitochondria regulates ROS generation. Impairment of one of these elements can trigger an uncontrolled increase ROS production (Aranda-Rivera et al. 2021). In UUO, high levels of NOX induced the overproduction of mitochondrial ROS generation which promotes oxidative stress. ROS overproduction by NOXs and mitochondria activates TGF-β1, which is a crucial mediator in promoting fibrosis. PCA treatment was able to prevent this cascade by improving mitochondrial function and inhibiting ROS production in human neuroblastoma cell line SH-SY5Y cells (Guo et al. 2019). PCA treatment also suppresses cisplatin-induced injury *in vitro* by blocking NOX-mediated oxidative stress (Gao et al. 2016). As redox signaling in renal fibrosis requires further exploration, further investigation of redox-sensitive processes should enhance the development of therapeutics targeting ROS and reduce fibrosis.

PCA has multiple pharmacological effects, including its potential in the prevention and treatment of diabetes and its complications (retinopathy, neuropathy, or nephropathy) (Jung et al. 2005; Lee et al. 2005). Prolonged hyperglycaemia in patients with diabetes activates aldose reductase, which metabolizes glucose in the blood to sorbitol, which then accumulates in the cells, causing eye, nerve, and kidney lesions (Hotta et al. 2012). PCA also has anti-inflammatory and anti-fibrotic effects on obstructive nephropathy (Yang et al. 2021). PCA inhibits aldose reductase in the polyol pathway (Jung et al. 2005; Lee et al. 2005). Therefore, the tubulointerstitial fibrosis inhibited by PCA in the diabetic nephropathy-activated polyol pathway might be a potential therapeutic strategy. Aldose reductase inhibitors have been reported to improve pulmonary and myocardial fibrosis (Li et al. 2015; Zhang et al. 2015; Wan et al. 2019). Future studies should investigate the effects of PCA on the polyol pathway in renal fibrosis.

EMT induces adult epithelial cells to acquire a mesenchymal or fibroblast phenotype. Moreover, EMT is considered one of the primary mechanisms that mediates renal interstitial fibrosis. Cytokine-driven EMT is the mechanism of the local generation of fibroblasts (Neilson 2006). Recently, EMT studies have provided new insights into the occurrence of epithelial dysfunction and tubulointerstitial fibrosis (Sheng and Zhuang 2020). Thus, although research has shown that fibroblasts play a critical role in fibrosis (Mack and Yanagita 2015; Sun et al. 2016; Kuppe et al. 2021), EMT, which accounts for a small proportion of fibrosis is still an indispensable mechanism for renal fibrosis, (Zeisberg and Duffield 2010).

Currently, there are no studies examining the anti-EMT and anti-fibrosis effects of PCA in renal proximal tubular cells, although other distinct compounds from natural products have been demonstrated to protect against renal fibrosis in the human renal proximal tubular cells HK-2 cells and in the UUO model such asalisol B 23-acetate (Chen et al. 2020c), poricoic acid A, and its derivative compounds (Wang et al. 2018, 2020; Chen et al. 2020a, 2020b). Additionally, some readily available drugs from our previous research have been reported to have anti-fibrosis effects in the UUO model. Rapamycin attenuates TGF-β1-induction which decreases UUO-induced renal fibrosis (Wu et al. 2006). Additionally, combining renin inhibitors valsartan with aliskiren A reduced UUO-induced renal fibrosis (Wu et al. 2010). In that these drugs have been used clinically, the above listed compounds are the ideal candidates to serve as positive controls. Although PCA and these drugs have positive effects on anti-renal fibrosis, the mechanism might be through a different pathway. Compare to PCA, rapamycin reduces renal fibrosis by inhibiting mTOR signalling, but this is not observed with PCA, thus, the mechanism of PCA still needs to be identified. Furthermore, a combination of PCA and rapamycin might yield an additive or synergistic response in renal fibrosis, through two different pathways of suppression. Additional work is required to examine this possibility.

## Conclusions

In the present study, PCA inhibited TGF-β1-induced EMT in NRK-52E cells as well as EMT-induced cell migration. In animal experiments, PCA suppressed UUO-induced EMT and renal fibrosis. Thus, PCA may be useful as a supplement or drug for preventing and treating renal fibrosis.
